# The Supporting Adolescent Adherence in Vietnam (SAAV) study: study protocol for a randomized controlled trial assessing an mHealth approach to improving adherence for adolescents living with HIV in Vietnam

**DOI:** 10.1186/s13063-019-3239-1

**Published:** 2019-02-28

**Authors:** Mary DeSilva, Cong Nguyen Vu, Rachael Bonawitz, Le Thanh Hai, Nguyen Van Lam, Le Thi Yen, Allen L. Gifford, Jessica Haberer, Dang Thuy Linh, Lora Sabin

**Affiliations:** 1 Westbrook College of Health Professions, University of New England, 716 Stevens Ave, Portland, ME 04103 USA; 2 Institute for Population Health and Development, No.18, Lane 132, Hoa Bang Str., Cau Giay District, Hanoi, 122667 Vietnam; 3 Center for Global Health & Development, Boston University School of Public Health, 801 Massachusetts Avenue, Crosstown 3rd Floor, Boston, MA 02118 USA; 4 National Hospital for Pediatrics, 18/879 La Thành, Đống Đa, Hanoi, Vietnam; 5 Department of Infectious Disease (ID), National Hospital for Pediatrics, 18/879 La Thành, Đống Đa, Hanoi, Vietnam; 6 Department of Health Law, Policy and Management, Boston University Schools of Medicine and Public Health, 725 Albany Street, Talbot T247W, Boston, MA 02118 USA; 7 Massachusetts General Hospital Global Health, 125 Nashua St, Suite 722, Boston, MA 02114 USA

**Keywords:** HIV, Antiretroviral therapy, Adherence, Adolescents, mHealth, Real-time feedback, Dose histories, Vietnam

## Abstract

**Background:**

The overall goal of the Supporting Adolescent Adherence in Vietnam (SAAV) study is to improve understanding of an adherence feedback mHealth intervention designed to help adolescents living with HIV (ALHIV) maintain high adherence to antiretroviral therapy (ART), critical to effective treatment. Specifically, we aim to: (1) conduct formative research with Vietnamese ALHIV and their caregivers to better understand adherence challenges and refine the personalized mHealth intervention package; and (2) assess the feasibility, acceptability, and efficacy of the intervention to improve ART adherence by implementing a randomized controlled trial (RCT).

**Methods:**

The study will utilize mixed methods. The formative phase will include 40 in-depth interviews (IDIs) with 20 adolescent (12–17 years)/caregiver dyads and eight focus group discussions with adolescents, caregivers, and clinicians at the National Hospital for Pediatrics (NHP) in Hanoi, Vietnam. We will also conduct 20 IDIs with older adolescents (18–21 years) who have transitioned to adult care at outpatient clinics in Hanoi. We will then implement a seven-month RCT at NHP. We will recruit 80 adolescents on ART, monitor their adherence for one month to establish baseline adherence using a wireless pill container (WPC), and then randomize participants to intervention versus control within optimal (≥ 95% on-time doses) versus suboptimal (< 95% on-time doses) baseline adherence strata. Intervention participants will receive a reminder of their choice (cellphone text message/call or bottle-based flash/alarm), triggered when they miss a dose, and engage in monthly counseling informed by their adherence data. Comparison participants will receive usual care and offer of counseling at routine monthly clinic visits. After six months, we will compare ART adherence, CD4 count, and HIV viral suppression between arms, in addition to acceptability and feasibility of the intervention.

**Discussion:**

Findings will contribute valuable information on perceived barriers and facilitators affecting adolescents’ ART adherence, mHealth approaches as adherence support tools for ALHIV, and factors affecting adolescents’ ART adherence. This information will be useful to researchers, medical personnel, and policy-makers as they develop and implement adherence programs for ALHIV, with potential relevance to other chronic diseases during transition from adolescent to adult care.

**Trial registration:**

ClinicalTrials.gov, NCT03031197. Registered on 21 January 2017.

**Electronic supplementary material:**

The online version of this article (10.1186/s13063-019-3239-1) contains supplementary material, which is available to authorized users.

## Background

In 2015, an estimated 1.8 million adolescents aged 10–19 years were living with HIV globally [1]. Despite effective treatments, data suggest that mortality is rising in HIV-infected adolescents compared to the general population [2], due in part to lack of access to care for youth in many resource-constrained settings [3]. Improving adherence to antiretroviral therapy (ART) remains critical for improving and maintaining treatment outcomes for adolescents living with HIV (ALHIV). Failure to maintain high adherence to ART, most typically defined as 95% or more [4–7], increases progression to AIDS and death [4, 8–14] and leads to drug-resistant strains of HIV, which can be transmitted to others [15–18]. The few studies of adherence to ART regimens in adolescents and young adults depend primarily on self-report but suggest suboptimal adherence as low as 41% [2], much lower than in adults [14] or younger children [19].

Sustaining high adherence to ART in adolescents presents unique challenges. Adolescence is characterized by a series of developmental changes that may include increased risk-taking behavior and decreased impulse control, culminating in the development of self as distinct from parents or family [20, 21]. As older children begin to assume responsibility for their medication, the array of developmental changes they experience, combined with complex and influential social and cultural factors [22], may test a child’s ability to adhere to treatment. How best to monitor adherence among adolescents is a particular challenge. Adherence measures in adolescent studies have primarily taken the form of self-report or other proxy indicators such as pill count and pharmacy refill [23–33], generally considered to be poor measures of true adherence in children [34–36] and adults [8, 37, 38]; only a few studies have included electronic drug monitoring (EDM), a more objective adherence measure [34, 35, 39]. These suggest that ART adherence is a challenge for adolescents, just as for adults.

Technology (mHealth) interventions may support adherence, if they are appropriately accurate, acceptable, and effective. Several mHealth interventions have been tested as adherence support tools in small samples of ALHIV in the United States, including phone reminders [23, 25, 26] and a web-based training program [27]. While initial findings are promising, all studies used self-reported adherence. Little research has been conducted on adherence, adherence challenges, or adherence-promoting strategies for adolescents in resource-limited settings. In particular, intervention studies using rigorous adherence measures are needed [40, 41].

Our recent mixed-methods study of adherence in adolescent ART patients in southern China found that wireless pill containers (WPCs) that monitored adherence electronically in real time were acceptable and feasible among youth, suggesting potential for use of such devices for both objectively measured adherence and real-time interventions for adolescents in an east Asian setting [42]. A previous intervention study utilizing WPCs with adult patients in China demonstrated that providing text message reminders triggered by missed doses increased ART adherence significantly [43]. We have accordingly designed the Supporting Adolescent Adherence in Vietnam (SAAV) study to build on this foundation of promising WPC research and add rigorous evidence regarding use of WPCs as an adherence support tool for ALHIV.

In Vietnam, where nationwide scale-up of ART began in 2005 [44], > 4300 children and adolescents living with HIV are currently on ART and the first surviving cohorts of pediatric ART recipients are transitioning to adult care [45]. Over 500 adolescents are on second-line therapy, raising concerns regarding their adherence, ART resistance, and the success of the country’s HIV treatment program. Thousands of adolescents will transition to care in adult outpatient clinics (OPCs) in coming years [45]. In a 2013 needs assessment of the transition to adult care in 21 OPCs in Vietnam, 42% of 1660 pediatric patients aged < 18 years were a single or double orphan, 62% had one or both parents on ART, and 50% lived too far from an OPC to access care as an adult [46]. In adolescents aged ≥ 15 years, 15% self-reported poor adherence and 22% were on second-line therapy, with both indicators increasing together with age. These figures portray a group of older youth in vulnerable circumstances, facing increasing challenges as they grow older.

SAAV will use mixed methods to develop and evaluate an adherence feedback mHealth intervention designed to help ALHIV in northern Vietnam maintain high adherence to ART as they near the age of transition to adult care. We believe this study will contribute valuable information on: (1) perceived barriers to and facilitators of adolescents’ adherence to ART; (2) mHealth approaches as ART adherence support tools for adolescents; and (3) factors affecting adolescents’ ART adherence. Findings will be useful to researchers, medical personnel, and policy-makers as they develop and implement adherence-promoting programs to support ALHIV as they approach transition to adult care, with relevance for youth with other chronic diseases.

## Methods

### Study aims and design

SAAV is a mixed-methods study to address four specific aims, comprising two phases. The specific aims are to: (1) examine perceived facilitators of and challenges to adherence among adolescent ART patients and their caregivers in Vietnam; (2) tailor an EDM-based real-time adherence support intervention package for adolescent Vietnamese ART patients, using direct input from adolescents into the development and individualization of the package; (3) assess the feasibility and acceptability of this personalized, mHealth approach to improving ART adherence among adolescents; and (4) generate preliminary data regarding the efficacy of the real-time feedback package on adherence, CD4 count, and HIV viral load (VL) in this patient population by implementing a small RCT.

Phase 1 (to address Aims 1 and 2) involves formative research, including focus group discussions (FGDs) with clinicians and adolescents, and in-depth interviews (IDIs) with adolescent/caregiver dyads and older adolescents who have transitioned to care in adult OPCs. Phase 2 (to address Aims 3 and 4) encompasses implementation of the prospective, randomized experiment to assess feasibility, acceptability, and impact of the intervention package on ART adherence and clinical outcomes. Participants will participate in the RCT described below and may participate in a follow-up IDI after the trial.

The study is informed by Self-Determination Theory (SDT), a psychological theory used to improve quality of patient motivation and, consequently, adherence behavior [47–49]. SDT informed one of the WPC-based interventions with adults in China, confirming correlations between key SDT constructs, such as competence, self-regulation, and support [50]. SDT has previously been used as a framework for interventions regarding behavior change with adolescents, particularly surrounding exercise regimens [51–53], as well as one study of adherence to asthma medication [54]. In baseline and endline surveys of the RCT, we will measure the component SDT constructs (perceived autonomy support, relative autonomy index, perceived competence, and relatedness) using validated scales. We have adapted the autonomy support scale to include perceived supportiveness of the WPC and triggered reminders, in addition to support from nurse and physician care providers. In applying the SDT model to adolescents, we expect that we may identify some important differences from adults, and therefore will measure additional items that emerged in our formative work with Chinese adolescents, including family routines, schedule interruptions, and self-efficacy. We will also measure other sources of social support, stigma, and depression, as these have been shown to impact adherence [55, 56].

The study has been approved by the Institutional Review Boards of the University of New England (reference 071316–001), the Institute for Population Health and Development (reference PHAD-2016/SAAV-01), and the Ethics Committee of the National Hospital for Pediatrics in Hanoi (reference VNCH-RICH-16-015).

### Research setting

We will conduct the study at the Infections Diseases clinic at the National Hospital for Pediatrics (NHP) in Hanoi, the central facility for diagnosis and research on all pediatric diseases in northern Vietnam. NHP provides outpatient services to approximately 450 HIV-infected children aged < 18 years, of whom about 200 are aged ≥ 12 years. The clinic is staffed with 12 physicians and 24 nurses. Patients visit the clinic monthly for medication refills, clinical exams, adherence assessments, and counseling for both the patient and caregiver. The NHP lab has capacity for HIV, CD4, and VL testing. Standard of care calls for CD4 testing every six months and VL testing every 12 months [57]. As soon as adolescents turn 18 years, they are required to transition to care in adult outpatient clinics in their home provinces. The IDIs with older adolescents in adult care will take place at one or more of the four outpatient HIV clinics in Hanoi that serve adults living with HIV.

### Research participants

*Adolescent participants* for Phase 1 (formative research) will be recruited from among HIV-positive patients aged 12–17 years who receive ART at NHP, along with their primary caregivers. All of these patients were perinatally infected. Adolescent participants for Phase 2 (RCT) will be recruited from the same NHP population. To be enrolled in either phase, adolescents must be on ART, in care at NHP (or graduated from NHP care in the case of older youth at adult OPCs), agree to follow study procedures, provide informed assent, and have their caregiver provide written informed consent. For the RCT, additional inclusion criteria will apply; participants in Phase 1 activities will be eligible to participate in the Phase 2 RCT. Adolescents must also expect to remain in care at the NHP for seven months minimum and be identified as having adherence challenges, defined by: detectable VL, CD4 < 700 in the last six months, or decline in CD4 in the last six months. Adolescents who are aged < 12 years or > 18 years, live outside the clinic catchment area, decline to provide informed assent, have a caregiver who declines to provide consent, or have a mental health condition sufficiently serious that they cannot understand informed assent, including the voluntary nature of participation, will be excluded.

*Adolescent participants* aged ≥ 18 years for Phase 1 (formative research) will be recruited from among HIV+ patients aged 18–21 years who have transitioned to care at OPCs in Hanoi. *Caregiver participants* for Phase 1 (formative research) must be identified as the child’s primary caregiver and will be recruited along with the adolescent patients at NHP selected for the IDIs or FGDs. *Clinician participants* from the Infectious Disease Clinic at NHP will be recruited for participation in Phase 1 (formative research) and all clinicians there will be eligible.

### Phase 1 (Formative) procedures

The IDIs with adolescent/caregiver dyads (20 dyads, for a total of 40 IDIs) will query challenges to adherence, including stigma, disclosure, school-related issues, caregiver relationships, and schedule interruptions. We will interview adolescents and caregivers separately, unless the caregiver requests to be present for the adolescent’s interview. The single FGD with clinicians (doctors/nurses) will probe adherence challenges, experiences caring for this population, and managing the transition to adult care. The FGDs with adolescents aged 12–15 years (six in total, three with girls, three with boys, for a maximum of 60 total participants) will explore adherence challenges, strategies for addressing challenges identified from the IDIs, and refinement of intervention package options. The IDIs with adolescents aged 18–21 years (20 in total, 10 with girls, 10 with boys) at adult OPCs will explore challenges to adherence and experiences with transition to care in adult OPCs.

For the IDIs and FGDs in Phase 1, we will purposively sample adolescent ART patients aged 12–17 years with the assistance of the clinicians at the NHP Infectious Disease Clinic, with a focus on convenience for the adolescent participants (e.g. timing of clinic visits, to minimize extra travel for patients), and sampling patients with a range of ages. All IDIs and FGDs will be conducted at NHP or adult OPCs in Vietnamese by trained interviewers in a quiet, private room, using a semi-structured question guide. All IDIs and FGDs will be digitally audio-recorded and supplemented with written notes. We expect each IDI and FGD to last approximately 1 h.

### Phase 2 (RCT) procedures

**Fig. 1:**
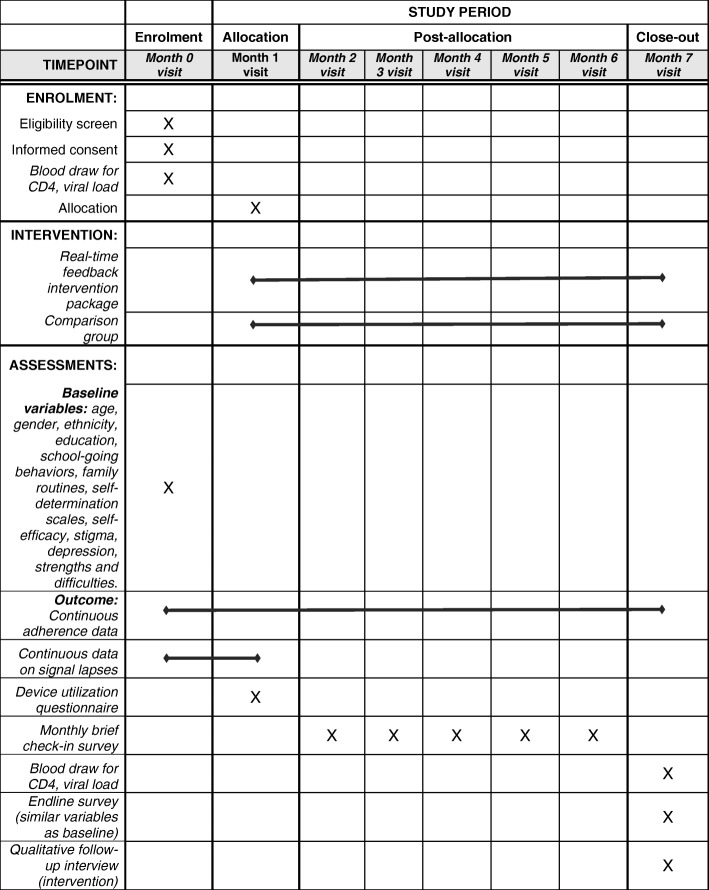
Schedule of enrolment, interventions, and assessments

**Fig. 2:**
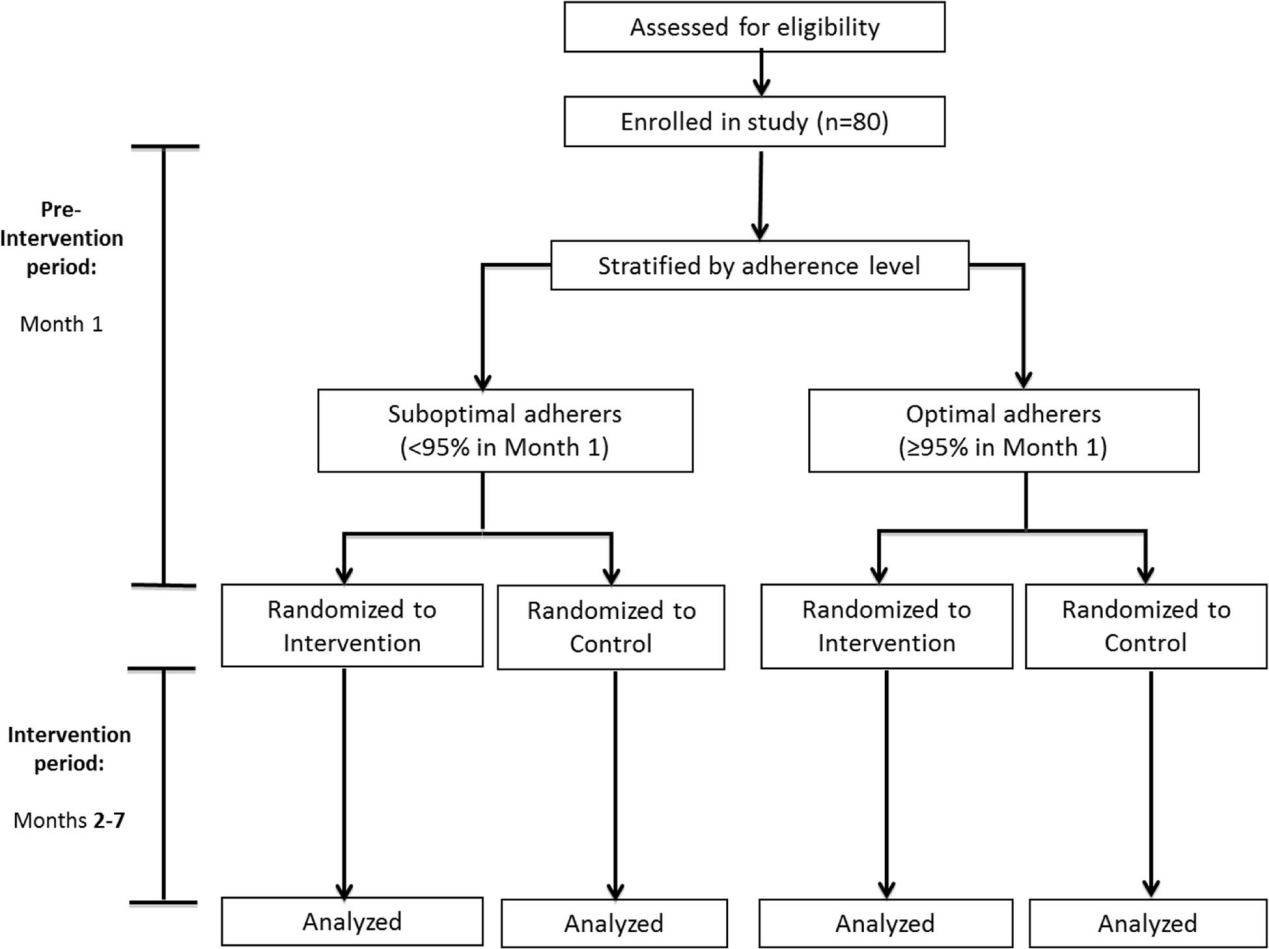
Study profile

We have used the Consolidated Standards of Reporting Trials (CONSORT) Statement as a framework for the development of the methodology for the RCT [58] and have also included a SPIRIT checklist (Additional file 1) and SPIRIT figure (Fig. 1). Each RCT participant will be enrolled for a total of seven months (one month of adherence monitoring; six months of intervention, Fig. 2).

### Intervention

The core intervention (real-time feedback intervention package) will utilize wireless technology to provide participants with: (1) real-time, personalized wireless reminder messages when ART doses are not taken by the scheduled dose time, via cellular signals sent by the WPC on openings to a server that will trigger messages; and (2) “feedback” on adherence behavior via monthly interactive counseling sessions informed by summaries of their previous month’s behavior. The core intervention will be personalized by each patient, who may tailor intervention features to suit his/her preferences by selecting: (1) reminders to the WPC (light or chimes) or to the patient’s or caregiver’s cell phone (text message or phone call) if the WPC is not opened by the scheduled dose time; (2) content of text messages from a menu of options; (3) “youth-friendly” WPC printouts for interactive counseling sessions; (4) additional counseling by clinic staff; and (5) a “buddy” system, by choosing someone to review the adherence report with him/her.

### RCT recruitment

Clinicians will briefly describe the study to all of their eligible adolescent patients aged 12–17 years and the adolescents’ caregivers in the course of patients’ regular clinic visits. The clinician will provide the adolescent/caregiver with the contact information of our study coordinator, who will be present at the study clinic at all times. If and when the adolescent/caregiver contacts the coordinator, he/she will arrange for a private face-to-face meeting, most likely at the clinic itself but elsewhere if preferred by the adolescent and his/her caregiver. At this meeting, the study coordinator will describe to the adolescent patient and his/her caregiver all aspects of the study, and what is entailed in study participation, including randomization, use of a WPC for seven months, provision of adherence, survey, and biological data, participation in adherence counseling sessions, and incentives. Those who wish to enroll and meet enrollment criteria (described above) will provide written informed consent (caregiver) and informed assent (adolescent) before the adolescent will be enrolled.

### Pre-intervention period and randomization (Month 1)

Once enrolled, each participant will be provided with a WPC (AdhereTech Gen2 device, AdhereTech, Inc., New York, NY, USA) and instruction on correct use. Each participant will select one tablet, either a single antiretroviral drug or in some cases, a combination tablet, to be stored in the WPC. While participants continue to receive care as usual, we will collect their adherence data using their WPCs. After one month, the study statistician will assess adherence over the month and randomize participants within adherence strata (optimal ≥ 95% vs sub-optimal < 95% on-time doses, defined by a ± 2-h window) to intervention or comparison group using a block randomization approach and one set of sequentially numbered opaque sealed envelopes containing computer-generated random assignments for each stratum. The statistician will provide the study coordinator with the participants’ assignments electronically via an encrypted data transfer system.

### Intervention period (Months 2–7)

The real-time feedback intervention will be implemented as described above. While comparison participants will not receive the reminders or adherence-informed counseling, they will continue to provide adherence data via their WPCs.

### RCT data collection procedures

Once enrolled, RCT participants will provide study data as follows:

1. *Sociodemographic data and SDT constructs*: upon enrollment, all participants will complete a baseline survey with the site coordinator which will cover: age; gender; ethnicity; education; school-going behaviors; family routines; scales for each of the SDT constructs; self-efficacy; stigma; a child depression index; and a strengths and difficulties index.
2. *Continuous adherence data in Months 1–7*: data on WPC openings will be transmitted continuously via 3G cellular network to a central server, which will automatically download data to a password-protected web-based database that investigators can access as needed for analysis. This system will provide adherence data for each participant. We will also collect data on the frequency of reminder messages sent to each participant by type of reminder, for intervention participants only.
3. *Continuous data on signal lapses*: during Month 1, we will investigate all WPC signal lapses lasting ≥ 48 h by phone call to the participant and determine whether a lapse was due to technical failure (battery failure, forwarder malfunction) or behavioral cause (missed dose, intentional non-use).
4. *Device utilization questionnaire*: we will administer a survey on adolescents’ experience using the WPC at the Month 1 visit for all participants.
5. *Monthly check-in questionnaire*: a brief questionnaire will be administered to all participants to collect data on: whether adherence counseling occurred (yes/no); who was responsible for WPC management; and the occurrence of any major life events (including status disclosure).
6. *Month 7 follow-up questionnaire*: a final survey will be administered to all participants at the end of the intervention period, similar to that completed at baseline, with the addition of a brief quantitative and open-ended questionnaire asking about experience with the WPC, including convenience of use, storage, and potential stigma/loss of confidentiality.
7. *Biological samples*: all participants will provide blood samples for CD4 count and VL tests at Month 1 (pre-randomization) and Month 7 (post-intervention period). Standard of care in Vietnam is CD4 count testing every three months and VL testing every six months free of charge to patients, but VL tests are rarely done due to cost. We will try to time enrollment to align with patients’ blood draw schedule such that, in general, no additional blood draws will be necessary for VL or CD4 tests, but some patients may be enrolled at a time when they are not due for a regular CD4 test; these patients will require an extra blood draw for the study at enrollment. All samples will be identified only by the patient’s study ID to ensure confidentiality. Data will be provided to the clinic for addition to participants’ charts in order to inform treatment. All extra testing costs will be paid for by the research project budget.

### Qualitative data

A subsample of 20 adolescent patient participants in the intervention arm will be selected to provide study information via audio-recorded IDIs in the last month of the intervention. The questions will focus on participants’ experiences with the WPC, receiving the cell phone reminder messages, receiving the device-generated adherence report, and counseling sessions, as well as their opinions about how the intervention as a whole and the personalized features of the intervention (which they chose) helped or failed to help them with their medication-taking behavior and views of approaches perceived as stronger support approaches. We will probe issues that relate directly to the SDT model, including: feelings of support; the degree to which aspects of the intervention changed patients’ sense of relative autonomy; and any changes in perceived competence. These data will enrich and help validate the quantitative data we will collect on these topics and inform our growing understanding of the relevance of the SDT model for the intervention in this population. We expect each IDI to take approximately 1 h to complete. The IDIs will take place in a private place at NHP to ensure confidentiality of information.

### Data monitoring plan

Because this is a behavioral study that does not involve any testing of medications, or any type of procedure that is outside the usual care provided to HIV-positive individuals, the study team’s role in monitoring patient safety will be limited to mandated adverse event (AE)/serious adverse event (SAE)/unanticipated problems reporting, alerting the site to unexpected test findings or conditions identified during participant participation, and reporting to the appropriate authorities any suspected abuse or maltreatment of adolescent participants. AEs will include clinical reactions to treatment and blood draws related to the study, as well as any reported stigma or physical and/or mental harm as a result of participation in the study, including disclosure of HIV status. All AEs will be recorded on designated forms by the on-site study coordinator and rated for both severity and seriousness. Any SAE or unanticipated event that occurs during the course of the study will be reported immediately to the PI and reported to the IRBs within one week of their occurrence. When necessary, the local study team can arrange for hospitalization and treatment at the nearest district hospital, in addition to referral to mental health services if required. Any events deemed by the study team to be possibly related to the study will be carefully reviewed and, if necessary, modifications to the protocol or informed consent will be made in order to protect the safety of study participants.

### Provisions to protect privacy and maintain confidentiality of data

We will ensure the confidentiality of adolescent patients to the greatest degree possible throughout the study. All interactions with participants will occur in closed rooms at the NHP clinic to ensure privacy. Data files, audio files, transcripts, questionnaires, and forms will be kept locked at all times when they are not in use by study personnel. Participants will each be assigned and identified only by a unique ID number, as will their caregivers, whose ID number will be associated with the adolescent for whom they provide care. All paper questionnaires, WPCs, other computer-based data files, and audio recordings will be accessible to named study personnel only, and only in forms without identifying personal identification of any kind other than the unique study ID number. Recordings will be destroyed after the patient completes the study or a final disposition is known. In addition, we will advise intervention participants to select a personal reminder that does not refer to HIV, to help prevent disclosure of HIV status if someone else reads the message. Because some adolescents may not have full understanding of their HIV diagnosis, interview guides and survey instruments will not refer to HIV explicitly but instead will use an appropriate Vietnamese translation of “your disease” when referring to HIV. Electronic data will all be maintained in password-protected files in encrypted folders and the file that links names and study numbers will be stored on computers in a locked office, with access limited to key study personnel. Study results and analyses presented in technical reports, manuscripts, and articles, including qualitative data, will either be presented in aggregate form, or with details removed to prevent identification of individuals.

### Data management and analysis

For all qualitative data, audio recordings will be transcribed, supplemented by written notes, translated into English by a bilingual translator. The English-language transcripts will then be checked by a second bilingual translator and coded and analyzed the transcripts using NVivo™ software. All survey data collected by research assistants for the RCT will be directly entered via tablet into Open Data Kit [59]. The study coordinator will coordinate data retrieval from the web-based server and will back up files weekly on a secure local server at the study office. The data manager will use standard procedures to conduct initial data cleaning, conversion into proper format for data analysis, and recoding. SAS version 9.4 will be used for all data cleaning, management, and quantitative analyses (The SAS Institute, Cary, NC, USA).

### Analytic approach, qualitative analyses (Specific Aims 1 and 3)

The analysis of the formative data collected in Phase 1 and the IDIs at the end of the Phase 2 intervention is by definition descriptive and will not be used to test study hypotheses formally. In Phase 1, our qualitative data will allow us to examine facilitators of and challenges to adherence as perceived by adolescent ART patients and their caregivers and clinicians. In Phase 2, IDIs will help to identify specific barriers to and facilitators of intervention effectiveness by tapping into the “grounded” experiences of adolescent patients who experienced the personalized intervention. For activities in each phase, we will use an iterative approach to: (1) identify themes; (2) build and apply a codebook; and (3) describe thematic characteristics, patterns, and relationships [60–62]. To promote validity and reliability, disconfirming evidence (e.g. negative cases) will be specifically identified. In Phase 1, we will compare adolescents’ responses to caregivers’ responses.

### Analytic approach (Specific Aim 3)

We will calculate the proportion-based key measures described above (enrollment, WPC use, technical failures), analyze reasons for refusal and drop-out, and supplement these findings with qualitative data from the Month 1 questionnaire, IDIs, and field notes. Feasibility and acceptability of *real-time feedback* will be indicated by proportions of: approached eligible patients who enroll; enrolled participants who complete the study; participants who report WPC use is convenient/easy; participants who report serious concerns with WPC or any intervention aspect; and intentional non-use of WPC as % of expected openings. If % technical failure as % of expected openings > 20%, this finding will suggest that the intervention may not be technically feasible.

### Analytic approach (Specific Aim 4)

In accordance with this study aim (to generate preliminary data regarding the efficacy of the real-time feedback package on adherence, CD4 count, and VL), this analysis will be non-confirmatory. We will assess differences between intervention and control in post-intervention proportions achieving optimal adherence, mean adherence, mean CD4, and proportions achieving UDVL, also stratified by optimal or sub-optimal adherence in the pre-randomization period. We will report the following comprehensive set of outcomes, including measures of effect, test statistics, and significance:

#### Adherence

- Mean adherence, comparing Month 7 to Month 0, and intervention versus control, and in each month of the intervention;
- % ≥ 95% adherence comparing Month 7 to Month 0, and intervention versus control, and in each month of the intervention.

#### Clinical markers

- CD4 count: comparison of mean CD4 count (SD) and change in CD4 between baseline and endline, and intervention versus control;
- viral load (VL): % UDVL after the intervention (Month 7), comparison between baseline and endline, and intervention versus control

### General approach

We will calculate descriptive statistics for all study variables (means, ranges, standard deviations, percent data missing, and frequencies, percentages for categorical variables). For adherence, a non-opening for a scheduled dose will be considered a non-adherent dose. Repeated openings within the same scheduled dose time will be excluded. Outcomes will not be imputed for patients who drop out. Patients who drop out before randomization will be excluded in experimental analyses. Sociodemographic and clinical characteristics from baseline will be compared between those randomized and those not randomized to assess representativeness of the final analytic sample, using Cochran Mantel–Haenszel χ^2^ tests for categorical variables and Student’s *t* tests for continuous variables. After randomization, we will compare dropout rates and characteristics of patients who drop out between study arms to assess potential for bias using the same statistical tests. All randomized participants will be included in analyses. We will perform both intention-to-treat (ITT) and per-protocol analyses.

For the main dichotomous outcome (% ≥ 95% adherence after the intervention [Month 7]), risk differences and 95% confidence intervals will be calculated. UDVL will be similarly treated as a secondary dichotomous outcome. We will then construct multivariate regression models to adjust for key baseline variables such as gender, using high adherence as the outcome, and intervention group as the primary predictor. Other variables found to be significantly associated with high adherence in bivariate logistic models will be added using forward-stepwise and forced entry techniques. We will also conduct stratified analyses based on gender and other attributes, although we may only have statistical power to detect trends. For continuous outcomes (change in adherence and CD4 count), we will use paired t-tests and General Linear Models to test for differences before and after intervention, then use similar multivariate approaches as described above. To examine interrelationships among adherence and the constructs of our theoretical model, stigma, and other psychosocial measures, we will generate bivariate Pearson correlations and conduct exploratory path analysis.

### Sample size and power considerations

The formative component in Phase 1 will include: IDIs with adolescent/caregiver dyads (20 dyads, for a total of 40 IDIs); one FGD with 8–10 clinicians; one FGD with 8–10 caregivers; up to six FGDs with adolescent patients (three with girls, three with boys, with 8–10 participants each; maximum 60 total participants); 20 IDIs with older adolescents aged ≥ 18 years receiving care at adult OPCs (10 with young women, 10 with young men). Total maximum qualitative participants will be 140: 40 IDIs with NHP adolescent patients/caregivers; 10 clinicians in FGD, 10 caregivers in FGD, 60 FGD participants aged 12–15 years; 20 IDI participants aged ≥ 18 years. These numbers are adequate to elicit a range of experiences of youth, their caregivers, and clinicians.

We have powered the RCT in Phase 2 to be able to detect meaningful changes in optimal adherence. We will recruit 80 adolescent patients, 40 per study arm, which will provide a minimum of 80% power at a two-sided alpha of *p* = 0.05 to detect specified differences in adherence between the two arms after intervention. Our estimates of outcome differences are based on our findings from our earlier CATS study with Chinese adults, in which we found that proportions achieving ≥ 95% adherence were 87.3% versus 51.8%, in intervention versus control patients after six months of the intervention [43]. We will have a minimum of 80% power to detect down to a 29 percentage-point difference in proportions achieving ≥ 95% adherence between arms after the intervention.

### Efforts to protect against standard risks of bias in unmasked trials

Although the nature of our intervention does not allow for blinding of trial participants or care providers, we have put into place the following protections. To protect against *selection bias*, we are using a stratified block randomization technique (as noted above) including allocation concealment via sequentially numbered, sealed, opaque envelopes. Due to the nature of the intervention, which involves clinician counseling, it is not possible to employ blinding, so our findings are at some risk of *performance bias*, although we are providing extensive training to care providers and study personnel to encourage them to standardize their interactions with all participants, with the sole exception of the intervention counseling component.

Our primary adherence outcomes for both intervention and control participants are measured using an electronic adherence monitoring device, an objective measure of adherence that does not involve human knowledge of allocation. Lab personnel who conduct CD4 and viral load processing are also not aware of allocation and have no direct contact with study personnel or participants. We believe that *detection bias* is therefore not of major concern to the study. As for *attrition bias*, we do not expect a great degree of loss to follow-up, withdrawals, or missingness for measures in this short, intensive study, but in addition to comparing background characteristics of those who withdraw or are lost to follow-up, to quantify the impact of missingness that may be non-random and possibly related to (for example) depression (non-ignorable missingness), we will conduct sensitivity analysis using ISNI methods. As noted above, we will conduct ITT and per-protocol analyses for comparison but will prioritize ITT in our reporting of findings.

### Dissemination

We have registered the trial in ClinicalTrials.gov. After data collection is complete, and within two years of completion, we will report results in ClinicalTrials.gov, including a flow chart of study recruitment and dropout, demographic and baseline characteristics of participants, primary and secondary outcomes (adherence, CD4, viral load), statistical test results, and AE information if applicable. After complying with NIH reporting requirements in ClinicalTrials.gov, we will undertake multi-faceted dissemination to make results available to a range of stakeholder audiences, including: (1) meetings in Vietnam with public health officials at all levels, interested patients, and the HIV community; (2) peer-reviewed publications in HIV, social science, and policy and health systems journals; and (3) presentations at national and international conferences, including the International Association of Providers of AIDS Care (IAPAC)’s annual International Adherence Conference. In the preparation of abstracts and publications, we will follow the criteria for authorship recommended by the International Committee of Medical Journal Editors (ICMJE); we do not intend to employ professional writers. If findings indicate, we will work with partners in Vietnam to plan a larger effectiveness study.

### Study principles

The protocol follows the *Standard Protocol Items: Recommendations for Interventional Trials* (SPIRIT) 2013 checklist (Additional file 1), although the reporting of the study’s findings will follow the *Consolidated Standards of Reporting Trials* (CONSORT) guidelines, using the extension for non-pharmacological trials [58].

## Operational issues and discussion

In addition to enhancing understanding of the issues adolescent patients face in order to better tailor support strategies for ART adherence, SAAV is designed to add to our understanding of the feasibility and effectiveness of wireless technology to improve adherence and viral suppression. At the time of submission of this protocol, the team was completing analysis of the Phase 1 qualitative data and enrolling patients for the RCT. During the preparatory period, the team encountered several issues that bear on the conduct of the study and which may be informative to other researchers involved in similar work with adolescents.

1. *Challenges of interviewing adolescents with caregiver present*. We had hoped to interview adolescents (both for the IDIs in the formative phase and the RCT) separately from their caregivers so that youth could be more comfortable in expressing themselves freely with study staff, but in the formative phase, we have encountered the situation that some caregivers prefer to stay in the interview room with their child. We realize that the study team cannot insist on separation, but we believe that some interview and survey data may be affected by a child’s eagerness to please a caregiver (who is present). For the RCT, the study team has devised several strategies to encourage (though never force) caregivers to wait outside the interview room. In all cases, we are documenting whether the caregiver is present for each study activity, in addition to noting how the child and caregiver related in cases when both were present.
2. *Reticence of many adolescents in formative phase*. We discovered that many of the adolescents in the IDIs were reluctant to talk in much detail about their medication-taking behavior, whether a caregiver was present or not. This finding is similar to our experience with adolescents of similar age in China [63]. Our local interviewers devised a series of icebreakers and other games to try to put the adolescents at ease, which has seemed to elicit some additional participation. Exploring adolescents’ general adherence challenges via peer engagement in a FGD format might be another beneficial strategy, perhaps with multiple meetings which could foster trust with facilitators and among participants.
3. *Increasing independence of older adolescents*. We are learning that the increasing independence of older youth in our study population has very real implications for the conduct of the research study. Many of the older adolescent participants (aged 15–17 years) regularly travel to the clinic on their own, which is less costly for the family, but makes it challenging to obtain caregiver consent and therefore to enroll these older youth in study activities. We recently obtained IRB approval to obtain verbal consent from caregivers by phone which we expect to facilitate RCT recruitment.
4. *WPC issues*. While many details had been worked out with the WPC vendor in advance and specified in the contract, additional features and services were requested during the preparatory period related to the use of Vietnamese language messages and specifics of the adherence report used in monthly counseling. These issues were resolved after discussion with the vendor and consultation with our local implementing partners who are experienced in using Vietnamese language with wireless technologies. Researchers should recognize that some logistics and technical details of any technology may be complex when used in global settings; teams should plan to allocate time for working out such matters whenever partnering with any vendor. Our team has had similar experiences with prior technology vendors.
5. *Identification of adolescents at high risk of low adherence*. The team is fully cognizant of the importance of using systematic criteria for identifying adolescents at risk of low adherence as eligible for the RCT. Despite limited preliminary VL data available to the team during the design phase of the study, during the set-up period, we learned that standard of care annual VLs are rarely obtained in northern Vietnam due to cost, so we relied on recent CD4 history (past three and six months) as the main proxies for low adherence risk. Although this not ideal, we were able to adjust the project budget in order to pay for baseline VL tests for all RCT participants which will help to contextualize our adherence findings. Similar future studies should anticipate such issues and include ample funding for dedicated lab testing.
6. *Incorporation into study of older adolescents who have already transitioned to adult care*. Those adolescents who have already transitioned to adult OPC care (aged 18–21 years) likely also experienced adherence challenges and perhaps even greater challenges than those of the NHP age group who are still in pediatric care. These youth are difficult to track in Vietnam, however, due in large part to frequent switching of cell phone numbers related to cell provider promotions. Although this was not explicitly part of our original study objectives, we thought it important to take advantage of our opportunity partnering with the NHP clinic to track some older adolescents who have recently graduated to adult care in order to learn about their experiences. We were able to modify the protocol to include additional IDIs with these older youth in the formative phase via a protocol amendment. We expect that findings from these interviews will provide valuable insight into the pediatric/adolescent HIV care continuum at the critical point of the transition to adult care.

Our experience to date implementing the initial components of the SAAV study highlights the challenges in conducting research in this population of adolescences, as well as the advantages of working closely with local partners to better understand treatment and care issues and respond quickly in terms of data collection modifications. If our hypotheses prove correct, we will advance learning in how to utilize powerful wireless technologies to help adolescent ART patients improve their medication-taking behavior as they approach care transition, with possible relevance for youth with other chronic diseases.

## Trial status

Protocol version number 1.2, approved by UNE IRB on 20 April 2017. Enrollment is active; RCT enrollment began on 15 May 2017 and is expected to be completed by 31 August 2017.

## Additional file

Additional file 1: SPIRIT 2013 Checklist: Recommended items to address in a clinical trial protocol and related documents. (DOC 124 kb)

## Abbreviations

AASC: Adolescent Adherence Support in China (study)
AIDS: Acquired immune deficiency syndrome
ALHIV: Adolescent living with HIV
ART: Antiretroviral therapy
CATS: The China Adherence Through Technology Study
DSMP: Data safety monitoring plan
EDM: Electronic drug monitoring
FGD: Focus group discussion
GPRS: General Packet Radio Service
HIV: Human immunodeficiency virus
IDI: In-depth interview
IRB: Institutional Review Board
NHP: National Hospital for Pediatrics
OPC: Outpatient clinic
PI: Principal investigator
RCT: Randomized controlled trial
SAAV: Supporting Adolescent Adherence in Vietnam (study)
SDT: Self Determination Theory
UDVL: Undetectable viral load
UNE: University of New England
VL: Viral load
WPC: Wireless pill container
